# Overview of Cancer Survivorship Care for Primary Care Providers

**DOI:** 10.7759/cureus.10210

**Published:** 2020-09-02

**Authors:** Sukesh Manthri, Stephen A Geraci, Kanishka Chakraborty

**Affiliations:** 1 Oncology, East Tennessee State University, Johnson City, USA; 2 Internal Medicine and Medical Education, East Tennessee State University, Johnson City, USA; 3 Medical Oncology, East Tennessee State University, Johnson City, USA

**Keywords:** cancer, survivorship, primary care

## Abstract

Survivorship care for a patient with cancer is often complex and requires a multidisciplinary approach. Cancer and its treatment can have late and long-term physical and psychosocial effects. After the acute and intense period of treatment and surveillance administered by oncology teams, cancer survivors slowly transition care to primary providers. Cancer survivors then enter into an extended phase of survivorship whether they are cancer-free, in remission, or living with cancer. In this phase, symptoms related to cancer and its treatment may vary over time. Developing a care plan can facilitate the transition of care between all providers taking care of cancer patients.

## Introduction and background

The American Cancer Society (ACS) estimates 1.8 million new cases of cancer will be diagnosed in 2020. A third of these patients will die from their malignancy [1]. According to statistics completed in January 2019, about 16.9 million people are currently alive in the United States after being diagnosed with cancer (this represents 5.0% of the population), a number that is expected to rise by ≥5 million by the year 2024 [2]. The cost of cancer care in the U.S. is rising and is expected to reach around $173 billion by 2020 [3].

The National Cancer Institute (NCI) definition is the most widely accepted: “An individual is considered a cancer survivor from the time of diagnosis, through the balance of his or her life." Family members, friends, and caregivers are also impacted by the survivorship experience and are therefore included in this definition [4]. Cancer survivors face multiple effects of both their cancer and its treatment, most of which can develop during treatment or manifest years after active therapy is concluded [5]. At least 50% of cancer survivors will have some consequences affecting their lives due to cancer and its’ treatment [6]. Major/frequent concerns for survivorship assessment includes cardiac toxicities, anxiety and depression, fatigue, pain syndromes, sexual dysfunction, sleep disorders, and the ability to maintain a healthy lifestyle. Many organizations have developed standards for the care of cancer survivors including the ACS, George Washington University, Institute of Medicine (IOM), the National Research Council, and the LIVESTRONG Foundation [7,8].

Key components of survivorship care plan include proper surveillance of cancer spread and recurrence, ongoing evaluation of the effects of cancer and its treatment on a patient, proper management of such effects, and coordination of care among all providers taking care of the cancer survivor [9]. The role of the primary care provider (PCP) in managing survivorship care is becoming even more important as the population of cancer survivors grows [10]. A 2009 survey conducted by the NCI and the ACS found that >50% of PCPs report that they administer survivorship care. PCPs with training on the effects of cancer and its treatment, who employ good communication in a co-management model with oncologists, were more likely to provide survivorship care [11]. However, the role of the PCP in survivorship care is not formally described [12]. This review will explore the common effects of cancer and its treatment including psychosocial effects, detection of secondary cancers, major long-term toxicities, and late side effects.

## Review

Psychosocial effects

Anxiety, depression, and post-traumatic stress disorders are the most common psychosocial consequences of cancer and its treatment. Together they affect 25-30% of cancer survivors [13]. Moreover, cancer survivors may develop altered social relationships, feelings of discrimination, and work-related difficulties. These may be related to fear of recurrence and changes in body image [14]. Assessment for psychosocial complications should be performed at least annually for all cancer survivors [15]. More detailed assessments should be given to patients at higher risk of psychosocial consequences (e.g., prior history of psychiatric disease, low social-economic status) [16]. Addressing these issues requires an inter-disciplinary approach that includes primary care providers, oncologists, psychiatrists, and others. This approach should employ psychopharmacological and psychological interventions to reduce psychosocial stressors and treat psychiatric illness on the cancer survivor and his/her caretakers [17].

Detection of second primary cancers and cancer recurrence

Surveillance for cancer recurrence, an integral part of survivorship care, is usually carried out for five years for most types of cancer. Depending on the type of primary malignancy, a combination of history-taking and physical examination coupled with laboratory and surveillance imaging are employed. Assessment and testing typically conform to a guideline-driven timeline (e.g., from the National Comprehensive Cancer Network [NCCN]). It is usually carried out by the oncologists and their teams. On the other hand, the detection of second primary cancers is usually carried out by the PCP. Second primary cancers in survivors are detected at a higher rate (2-30%) depending upon initial primary cancer type, compared to the general population [6]. Explanations for this observation include predisposing genetic disease, prior exposure to chemotherapy and/or radiotherapy, and the shared causative factor (i.e., a risk factor for both types of cancer) [18]. Most recently, the ACS recommended screening for secondary malignancies similar to screening for initial cancers in the general population. PCPs should carry a discussion about the risks and benefits of a particular screening modality with each patient. Some survivors may require screening for secondary malignancy according to recommendations that differ from the general population. One example is female carriers of Lynch syndrome who require cancer screening at the age of 35 (lifetime increased risk for cancers - colorectal, endometrial, urinary tract, pancreatic or biliary, ovary, gastric, small intestine and others). Another example is female survivors who received thoracic radiation as part of Hodgkin’s lymphoma treatment will require breast cancer screening with MRI. Communication between treating oncologists and primary providers is required in such patients [19].

Long-term toxicities and late side-effects

Cancer survivors are at high risk to develop complications secondary to cancer and its treatments. The ACS, American Society of Clinical Oncology (ASCO), and the NCCN periodically publish survivorship care guidelines. Table 1 summarizes common complaints of cancer survivors and the recommended approach for addressing them. Moreover, some cancer patients receive long-term (years) treatment with a cytotoxic agent, hormonal treatment, androgen deprivation therapy, targeted therapy, or immunotherapy. Primary care physicians should be aware of the common toxicities of the most commonly-used cancer therapies (Table 2).

**Table 1:** Common cancer and treatment-related long-term and late effects with the recommendation ENT: Ear, Nose & Throat, BFI: Brief Fatigue Inventory, MDASI: MD Anderson Symptom Inventory, NSAID: nonsteroidal anti-inflammatory drug, DEXA: dual-energy x-ray absorptiometry

Problem	Common Symptom	Related Malignancy	Recommendation
GI Issues	Obstruction	Colon, rectal, and anal malignancies	Discuss bowel habits, rectal bleeding, and sphincter dysfunction
	Rectal bleeding/ulcers		Evaluate for incisional hernia
	Challenges with ostomy care		Refer to an appropriate specialist
Dental/Oral	Loss of taste	Head and neck	Adequate oral hygiene
	Xerostomia hyposalivation		Evaluate for nutritional deficiencies
	Dental caries		Referral to a specialist (ENT, dentists)
Fatigue (rating in a scale of 0-10)	Tiredness	Common to most	Use of an assessment tool (MDASI, BFI)
			Optimize nutrition and physical activity
			Assess for causative etiology: anemia, thyroid dysfunction, sleep disorder
Pain	Somatic	Common to most	Assess using pain scale and comprehensive history and exam
	Neuropathic		Offer intervention (NSAID, antidepressants, anticonvulsants, opioids, physical activity)
			Refer to a specialist once etiology is determined
Lymphedema		Breast	Offer compressive garment
			Refer symptomatic patients to a specialist
Bone Health		Breast, prostate, and others	Baseline DEXA scan
Cognitive Dysfunction	Memory changes	Cranial radiation (lung, breast, melanoma)	Assess by asking if the patient is experiencing cognitive dysfunction
	Difficulty multitasking		Assess for contributing factors (medications, mood disorder, symptoms burden)
			Refer symptomatic patients for neurocognitive testing
Cardiotoxicity	Chest pain	Breast, gastric, lung	Assess symptoms of heart failure, modifiable and non-modifiable cardiac risk factor
	Shortness of breath		Consider echocardiogram in a symptomatic patient
	Swelling of legs		Consider referral to a specialist
			Management depends on the stage of heart failure according to ACCF/AHA
Sexual Health	Erectile dysfunction	Pelvic radiation (prostate, bladder, rectal)	Assess by using the Sexual Health Inventory for Men
	Vaginal dryness	Hormonal treatment (breast)	Assess using the Brief Sexual Symptom Checklist for woman
	Interest changes	Androgen deprivation therapy (ADT) - prostate, breast	Obtain detailed medical, psychosocial, and oncologic histories and physical exam
			Obtain appropriate laboratory studies
			Avoid testosterone use in men receiving ADT

**Table 2:** Common toxicities of the most commonly-used cancer therapies VEGF: vascular endothelial growth factor, HER-2: human epidermal growth factor receptor 2, mTOR: mammalian target of rapamycin, PIK3: phosphoinositide 3-kinase, CTLA: cytotoxic T-lymphocyte-associated protein, PD-1: programmed death-1

Therapy	Category	Serious side effect
Cytotoxic		
Anthracycline (doxorubicin)	Antibiotic	Cardiotoxicity
Taxane (paclitaxel, docetaxel)	Mitotic inhibitors	Peripheral neuropathy
Platins (cisplatin, carboplatin)	Alkylating agent	Peripheral neuropathy, ototoxicity
Bleomycin	Inhibition of DNA synthesis	Pneumonitis, pulmonary fibrosis
Capecitabine	Antimetabolite	Hand-foot syndrome
Vincristine	Mitotic inhibitors	Peripheral neuropathy
Cyclophosphamide	Alkylating agent	Secondary malignancy
Etoposide	Topoisomerase II inhibitor	Secondary malignancy
Monoclonal antibodies		
Bortezomib	Proteasome inhibitor	Peripheral neuropathy
Bevacizumab	VEGF inhibitor	Hypertension, thromboembolic events
Trastuzumab	HER-2 inhibitor	Cardiotoxicity
Targeted therapy		
Everolimus	mTOR inhibitor	Pneumonitis, hyperglycemia and, dyslipidemia
Nilotinib	Tyrosine kinase inhibitor (TKI)	QT prolongation
Vemurafenib	B-Raf enzyme inhibitor	Squamous cell carcinoma
Sorafenib	TKI	Hemorrhage, hepatoxicity
Ibrutinib	Bruton's tyrosine kinase (BTK) inhibitor	Peripheral edema, atrial fibrillation, bruising
Idelalisib	PIK3 inhibitor	Colitis, hepatitis, pneumonitis
Endocrine treatment		
Tamoxifen	Selective ER modulator	Thromboembolism, endometrial cancer
Aromatase Inhibitors		Osteoporosis, vaginal dryness, arthralgia
Androgen-deprivation therapy		sexual and cardiovascular dysfunctions Osteoporosis
Immunotherapy		
Ipilimumab	anti-CTLA	Colitis
Nivolumab	anti–PD-1	Hypothyroidism

The financial burden of cancer care

Based on NCI cancer trends progress reports, national expenditures associated with cancer have been steadily increasing in the United States. Cancer is one of the most expensive medical conditions to treat in the United States. Medical care expenditures for the care of cancer survivors accounted for an estimated $137.4 billion in the United States in 2010 and are expected to rise to as high as $173 billion by 2020. National expenditures were largest for female breast, colorectal, prostate, lymphoma, and lung cancers, reflecting the prevalence of the disease, treatment patterns, and costs of care. Estimates of national expenditure in 2018 for breast cancer care is $19,700 million, colorectal is $16,630.9 million, prostate is $15,299.2 million, lymphoma is $14,626.7 million, and lung cancer is $14,185.5 million. As the population ages, cancer prevalence will increase even if cancer incidence rates remain constant or decrease somewhat. Costs are also likely to increase as new, more advanced, and more expensive treatments are adopted as standards of care. Financial toxicity in cancer survivors may lead to not taking medications as directed, having a lower quality of life, and debt and bankruptcy. Cancer survivors may have financial problems many years after they are diagnosed due to ongoing payment for ongoing cancer treatment or care for late effects from their treatment [20].

Special considerations like the impact of pandemics like COVID-19 on cancer survivors

The coronavirus disease 2019 (COVID-19) pandemic has raised challenges and increased the complexity of cancer care. Important questions include balancing the risk from treatment delay versus harm from COVID-19, limiting the use of immunosuppressive cancer treatments whenever possible, mitigating appropriately and fairly allocating limited healthcare resources. Limited healthcare resources can lead to delays in care delivery, screening, and surveillance. Patients with long-term immune suppression may be at increased risk of infection. Evolving data indicate a high rate of severe disease and mortality from COVID-19 in patients with lung cancer. The impact on cancer patients cannot be quantified at this moment and will be determined in upcoming months. 

Specific tumor types

Testicular Cancer

Testicular cancer (TC) survivors present a challenging population. The incidence of TC in the U.S. has increased in the past 20 years in men aged 18 to 39 years [21]. Chemotherapy is the mainstay of treatment. A combination of cisplatin, bleomycin, vinblastine, and ifosfamide is commonly used. The relative 10-year survival rates approach 95% for all patients with TC [22]. Survivors have a 1.5-4.0-fold increased risk for developing a second malignancy. The risk is even higher (a 5.9-fold increase) in patients who had received radiotherapy and chemotherapy [9]. Although there is no consensus on surveillance in this group, many experts recommend that TC survivors undergo cancer screenings, which is applicable to the general population [18]. Moreover, TC survivors are at an increased risk for cardiovascular disease and pulmonary toxicity. Approximately 7-21% of TC patients who receive bleomycin will develop pulmonary toxicity. If TC survivors require surgery any time after treatment, a thorough cardiopulmonary evaluation is recommended [23,24]. Finally, hypogonadisms and infertility are common among TC survivors. Aging, chemotherapy, orchiectomy, and radiotherapy all contribute to infertility. Young men who have not completed their family are required to be counseled for sperm preservation. This adds to the cost of the treatment as the patient needs to pay for the preservation and maintenance of the sample. Low testosterone levels and increased serum concentrations of luteinizing hormone and follicle-stimulating hormone are commonly detected. The risk for testosterone deficiency is 3.3- to 5.2-fold higher in TC survivors than in the general population. Testosterone replacement is often attempted, but no data support this approach [25]. All cancer survivors should undergo continuous efforts to promote a healthy lifestyle, including consuming a balanced diet and smoking abstinence.

Head and Neck Cancer

The structure and function of the head and neck allow for many critical processes including swallowing, speaking, airway patency maintenance, and taste. Head and neck cancer (HNC) patients typically require intensive multi-disciplinary care from otolaryngologists, medical and radiation oncologists, and other healthcare providers (dentists, nutritionists, gastroenterologists, etc). Early-stage and node-negative HNC can be treated with surgery or radiotherapy alone, while locally advanced HNC require chemotherapy (platinum-based) as part of the treatment plan. Functional loss, vascular complications, and hypothyroidism are frequently discovered in HNC survivors. The incidence of esophageal strictures leading to dysphagia is 20-30%, with 82% of these presenting within the first post-treatment year [26]. Discordance often exists between the clinicians and the patient’s judgment of dysphagia. Aspiration presenting with chronic cough, frequent pneumonia, and weight loss is another frequent complaint by HNC survivors. However, up to 50% of chronic aspirators can be silent and are only detected by swallowing studies [27]. There is a role for cancer rehabilitation, including speech and language therapists with a therapy consisting of swallowing exercises and maneuvers, neuromuscular stimulation, and education regarding the importance of lifestyle modification (eg, changing the food consistency to prevent aspiration) can help prevent worsening (or more significant) morbidity. Carotid artery compromise may also occur, though routine ultrasound screening is not supported by data [28]. Dental caries secondary to xerostomia is also common (24%), and nutritional status and oral health should be assessed routinely [29]. Alcohol-free rinses can be used to rehydrate the oral mucosa and prevent crusting. Avoidance of caffeine, spicy foods, and tobacco should be encouraged in HNC survivors [27]. Conversely, hypothyroidism is a late post-treatment complication; thyroid stimulating hormone (TSH) should be checked at six- to 12-month intervals as recommended by NCCN. Finally, jaw pain or swelling may signal osteonecrosis. Early conservative management with daily chlorhexidine gluconate and broad-spectrum antibiotics coupled with early referral to oral surgeons can prevent osteonecrosis of jaw from progressing [30].

Lung Cancer

Lung cancer, the most common cause of cancer death in the U.S., is common, with the majority of patients diagnosed after age 60 years. ACS 2013 estimated 412,230 lung cancer survivors. The IOM recommends developing care plans for cancer survivors. This is particularly more valuable to lung cancer survivors giving the complexity of lung cancer treatments. The care plan, prepared by the oncology team, should include a summary of treatments provided including possible side effects and formal follow up plans [31]. Care plans would assist in the sharing of knowledge and patients care among oncologists and primary providers. Lung cancer patients are most often (though not always) long-term smokers and are exposed to various treatment modalities (medical therapy, radiation, and surgery), placing survivors at particularly high risk for developing secondary malignancies (largely owing prior exposure to causative factors and exposure to therapy). Risk of lung, larynx, and bladder secondary malignancies remains elevated even 10 years after initial diagnosis [32]. Respiratory complaints (cough, dyspnea, and reduced exercise tolerance) are among the most common complaints among lung cancer survivors. Survivorship care should focus on counseling on smoking cessation, optimizing treatment of chronic obstructive lung disease (COPD, a frequent co-morbid condition), and promoting an overall healthy lifestyle [33]. Physicians should realize it is never too late to council lung cancer patients on smoking cessation. Uncontrolled cancer-related pain is reported in 45% of cancer patients [34]. Pain is commonly associated with anxiety and depression leading to disability and impaired quality of life (QOL) among lung cancer survivors. Post thoracotomy pain syndrome affects 80% of lung cancer patients receiving surgical resection. Thirty percent of lung cancer survivors had chronic, mild post-thoracotomy pain lasting four years post-resection. Other factors impacting QOL in cancer survivors are distress and fear of recurrence. Distress is reported in 25-40% of cancer survivors while fear of recurrence ranges from 5-89%. Both factors are more pronounced in lung cancer survivors. Distress and fear of recurrence vary over time in cancer survivors and may be triggered by the onset of unexplained symptoms. Social support and coping strategies can have a positive impact on QOL [35]. 

Colon Cancer

The overall five-year survival rate for colorectal cancers (CRC) has increased to ~65% in recent years. Moreover, the survival of stage IV CRC has also improved with the majority of patients receiving maintenance therapy for an extended period of time [36]. Bowel dysfunction is common among CRC survivors. Up to 50% of patients may report diarrhea lasting more than four weeks and affecting lifestyle, requiring anti-diarrheal agents. Chemotherapy-induced peripheral neuropathy (C-IPN) is also common (40%), especially in patients receiving oxaliplatin (>900 mg/m2 cumulative dose). Only about half of these patients will experience a total resolution of symptoms [36]. Patients with diabetes and prior alcohol abuse are particularly susceptible. Unfortunately, no data support any preventive measures. Duloxetine, 30 mg daily for one week followed by 60 mg daily for four weeks, appears to be beneficial in reducing neuropathic symptoms [37]. Urogenital and sexual dysfunction is seldom acknowledged as a long-term complication, especially in women receiving radiotherapy for rectal cancer [38]. Dyspareunia may be reduced with vaginal lubricants. Male CRC survivors with erectile dysfunction may benefit from oral phosphodiesterase-5 inhibitors [39]. Finally, bladder dysfunction should be assessed to determine whether symptoms are from a hypo-contractile vs hypoactive bladder, and treatment should be based on such assessment.

Prostate Cancer

Prostate cancer (PC) survivors share common issues with breast cancer survivors (see below). The majority of men diagnosed with PC are 60-70 years old. More than 90% of afflicted patients present with the local or regional disease for which the expected five-year survival approaches 100% [40]. Men with local or regional PC who had received radiotherapy and are receiving only androgen deprivation therapy (ADT) are an important subset of PC patients. ADT increases the risks of cardiovascular disease, osteoporosis, and sexual dysfunction. Clinicians should follow the same screening and prevention protocols for cardiovascular disease as recommended for the general population. Men on ADT should have a DEXA scan (dual-energy x-ray absorptiometry) and FRAX (world health organization fracture risk assessment tool) score calculated. Bisphosphonate and denosumab are FDA-approved for PC patients on ADT who qualify for osteoporosis treatment. Sexual dysfunction can be assessed by screening tools such as the Sexual Health Inventory for Men [41]. A trial of phosphodiesterase type 5 inhibitors, urologic consultation, and/or psychotherapy evaluation are potential interventions. After definitive radical prostatectomy is completed, prostate-specific antigen (PSA) serum concentrations should be undetectable by two months after surgery [42]. After definitive radiation therapy (RT) with or without ADT is completed, PSA increase by 2 ng/mL or more above the nadir PSA is the standard definition for PSA persistence/recurrence and a recurrence evaluation should be considered. The same conception is applied when PSA has been confirmed to be increasing after RT even if the increase above nadir is not yet 2 ng/mL, especially in candidates for salvage local therapy who are young and healthy [43]. NCCN recommends measuring serum PSA every six to 12 months for the first five years after treatment, then annually and an annual digital rectal exam. Any recurrence or persistence in PSA or new mass on palpation should prompt referral to primary treating specialist for consideration for surgery or radiation based on initial definitive therapy [44].

Breast Cancer

Almost four of every 10 female cancer survivors had breast cancer (BC) as their primary malignancy, with an estimated 3.6 million breast cancer survivors in the U.S. as of 2016-2017. Breast cancer treatment has improved significantly in the past decade, reflected in a five-year survival rate of almost 90% among all breast cancer patients [45]. The treatment approach for BC varies with the patient’s clinical-stage, menopausal status, and tumor-specific characteristics (hormone receptors, HER-2 neu status). Treatment may include surgical resection (lumpectomy or mastectomy), radiotherapy, chemotherapy, and/or hormonal treatment. Some patients may require adjuvant endocrine therapy (aromatase inhibitor by suppressing plasma estrogen levels or selective estrogen receptor modulator by competitive antagonism of the estrogen receptor) for the woman with hormonal receptor-positive BC for a period of five to 10 years. BC survivors are at a high risk of cardiac toxicity. Prior exposure to radiation, anthracyclines, trastuzumab, and aromatase inhibitors can all contribute to progressive cardiac dysfunction [46]. Clinicians should assess symptoms of cardiac disease, monitor lipid levels, and prescribe preventive measures including therapeutic lifestyle changes. Also, exposure to these therapies, combined with increasing age, lack of exercise, vitamin D deficiency, and low serum calcium levels, all increase the likelihood of osteoporosis in BC survivors. A repeat DEXA scan testing is advised one or two years after starting treatment for a woman on an aromatase inhibitor or who experiences ovarian failure secondary to chemotherapy when the results are likely to influence clinical management. Calcium (1200 mg daily) and vitamin D (600 IU daily) should be prescribed while BC survivors are receiving an aromatase inhibitor per ACS guidelines [47]. The PCP should assess for body image concerns which affect 31-67% of BC survivors, and an even higher incidence is seen among younger women. Clinicians should assist patients in obtaining prosthetic devices, wigs, and other resources to improve their body image. Surgical interventions (reconstruction/prosthetic implantation) and mental health referral may be helpful in select cases.

## Conclusions

Caring for cancer survivors is a collaborative effort. Most cancer patients will have a close follow-up with their primary specialist during the intense period of cancer treatment. Survivors may slowly transition care from their subspecialist to their PCP. The IOM recommends that treating specialists provide survivorship care plans, which include a treatment summary and follow-up recommendations, to the PCP when the transition of care begins. PCPs should understand the most common long-term and late effects of common cancers and their treatments. They should also have access to common survivorship resources.
